# Evaluating the Lipid Quality of Yellowfin Tuna (*Thunnus albacares*) Harvested from Different Oceans by Their Fatty Acid Signatures

**DOI:** 10.3390/foods10112816

**Published:** 2021-11-16

**Authors:** Valentina F. Domingues, Mário Quaresma, Sara Sousa, Mónica Rosas, Breixo Ventoso, Maria Leonor Nunes, Cristina Delerue-Matos

**Affiliations:** 1 REQUIMTE/LAQV-GRAQ, Instituto Superior de Engenharia do Porto, Instituto Politécnico do Porto, 4200-072 Porto, Portugal; vfd@isep.ipp.pt (V.F.D.); sara.sousa@graq.isep.ipp.pt (S.S.); monra@isep.ipp.pt (M.R.); cmm@isep.ipp.pt (C.D.-M.); 2 Centre for Interdisciplinary Research in Animal Health (CIISA), Faculty of Veterinary Medicine, Lisbon University, Pólo Universitário Alto da Ajuda, 1300-477 Lisboa, Portugal; 3 PhD Student of Health Science Program (UCAM), Universidad Católica San Antonio de Murcia, Campus de los Jerónimos, Guadalupe, 30107 Murcia, Spain; breixo.ventoso@grupofrinsa.com; 4 CIIMAR, Interdisciplinary Centre of Marine and Environmental Research, University of Porto, Terminal de Cruzeiros do Porto de Leixões, Av. General Norton de Matos S/N, 4450-208 Matosinhos, Portugal; nunes.leonor@gmail.com

**Keywords:** fatty acids, yellowfin tuna, *Thunnus albacares*, fatty acid ratios, lipid quality indices, total lipid

## Abstract

The aim of this study was to evaluate the influence of the fishing location on yellowfin tuna’s (YFT; *Thunnus albacares*) white muscle total lipid (TL) content and fatty acid profile. The comparison included 45 YFT loins, equally divided between the Atlantic, Indian and Pacific Oceans. The ocean had no significant influence on YFT TL content, total PUFA and total n-3 PUFA (*p* > 0.05), averaging 1.09 g/100 g of muscle, 229.2 and 192.8 mg/100 g of muscle, respectively. On the other hand, the YFT harvested on the Indian Ocean displayed significantly higher contents of both SFA and MUFA totals than the Atlantic Ocean counterparts (*p* < 0.05), while the YFT harvested in the Pacific Ocean presented intermediate values, not differing significantly from the other two origins. The YFT from the Indian and Pacific oceans held twice the n-6 PUFA content recorded in the Atlantic YFT (44.1 versus 21.1 mg/100 g of muscle). Considering the recommended daily intake of EPA plus DHA is 250 mg, the consumption of 100 g of YFT from the Atlantic, Indian and Pacific Oceans would provide 149.2 mg, 191.8 mg or 229.6 mg of EPA plus DHA, representing 59.7%, 76.7% or 91.8% of the recommended daily intake, respectively.

## 1. Introduction

The total world fishery production, in 2018, was estimated to be 178.5 million tonnes, of which 7.9 million tonnes were of tuna [1]. Among tunas, seven species are of global commercial importance, namely: albacore (*Thunnus alalunga*), bigeye (*Thunnus obesus*), skipjack (*Katsuwonus pelamis*), yellowfin (*Thunnus albacares*) and three species of bluefin tuna (Atlantic bluefin (*Thunnus thynnus*), Southern bluefin (*Thunnus maccoyii*) and the Pacific bluefin (*Thunnus orientalis*) [1,2]. Data from 2016, the most recent data displaying the amount of tuna harvested by species, revealed that skipjack tuna and yellowfin tuna (YFT) represented respectively 54.7% and 28.3% of total catches, and these two tuna species support the canning industry of tuna around the world.

Tuna is an important commodity worldwide. In 2019, the European Union (EU) imported 787,613 tonnes, 72% canned tuna and 28% frozen tuna, with a market value of 3.17 billion € [3]. Canned tuna is the third most consumed seafood worldwide, with an estimated consumption of 0.96 kg/per capita [4]. The apparent per capita consumption of tuna in the EU reaches 3.14 kg, which represents about 13% of the total consumption of fish [3].

Presently, most of the canned tuna imported by the EU (75%) consists of skipjack tuna, but the YFT is gaining popularity among consumers, due to its rich taste and excellent texture. Therefore, the market share of yellowfin tuna is expected to boost the segment growth over the 2020–2027 period [5].

Fish and seafood products have a high nutritional value, since they present high protein quality with a low caloric density. Despite their low fat content, they supply long chain polyunsaturated fatty acids (PUFA), as the eicosapentaenoic and docosahexaenoic acids (EPA and DHA, respectively), which are required for human regular growth and development [6], and exert anti-inflammatory and neuroprotective effects [7,8]. Moreover, fish and seafood products are a good source of minerals and vitamins [9]. Lipids are the predominant source of energy for fish and are stored in fat depots in different tissues. Their contents are known to vary in relation to the reproductive cycle and tunas allocate a substantial amount of energy into gamete production [10]. However, fish species inhabiting tropical and subtropical regions, as YFT does, benefit from relatively constant environmental conditions, being able to fulfil their energy requirements for reproduction mainly through feeding with little mobilization of stored lipids. For that reason, YFT is classified as an income-capital breeder [11]. These species do not reveal considerable changes in their fat depots [10,11]. Moreover, lipid mobilization occurs in liver rather than in white muscle [11], allowing the YFT white muscle to present similar content of total lipids and sensorial characteristics throughout the year.

The YFT is a species with great economic importance, since its meat is quite versatile and can be consumed raw, cooked, smoked, and canned [12]. The aim of this study was to evaluate YFT’s white muscle lipid content and its fatty acid profile, obtained from specimens harvested in the Atlantic, Indian and Pacific Oceans.

## 2. Materials and Methods

### 2.1. Chemicals

Sodium hydroxide and anhydrous sodium sulfate were obtained from Pronalab (Lisbon, Portugal); boron trifluoride-methanol (BF3) (14% in methanol), Supelco 37 Component FAME Mix and butylated hydroxytoluene (BHT) (≥99%) were from Sigma-Aldrich (St. Louis, MI, USA); dichloromethane, methanol and n-hexane (99%) were from Merck (Darmstadt, Germany); Tridecanoic acid (C13:0) was from Fluka (Buchs, Switzerland) and sodium chloride (99.5%) was from Panreac (Barcelona, Spain).

### 2.2. Sampling

A total of 45 samples of yellowfin tuna (*Thunnus albacares*; YFT) specimens were used in this trial. They were harvested in the Atlantic (*n* = 15), Indian (*n* = 15) and Pacific (*n* = 15) oceans, within the FAO fishing areas 34, 51 and 71, respectively. The YFT weight, fishing year/period and FAO’s fishing zone are depicted in Table 1.

The YFT specimens were delivered frozen to below −20 °C to Frinsa’s frozen storehouse between July and December of 2020. The selection of the YFT lots to use in the study and the collection of the specimens was performed in January of 2021.

Among Frinsa’s frozen stored yellowfin tuna, 15 specimens from each ocean were randomly selected and defrosted. The YFT’s muscle portion, white muscle exclusively, was obtained from the dorsal portion, positioned between the head and the first dorsal fin.

The YFT muscle was vacuum-packed and stored under refrigeration during transport to the laboratory. The muscle was then equally divided into three portions, by perpendicular cuts to its craniocaudal axis, and the middle portion was used to evaluate the lipid content and the fatty acid profile. On the day of arrival to the laboratory, the middle portion was cut in slices and then into small pieces, which were blended in a domestic food processor (Thermomix TM5), stored in refrigeration (<5 °C) over night, and processed the day after.

### 2.3. Intramuscular Total Lipid Content and Fatty Acid Composition

The total lipid content (TL) was extracted according to the Folch procedure [13], using dichloromethane rather than chloroform [14]. The TL determination was performed in duplicate and the results are expressed as g lipid/100 g/sample.

Fatty acid methyl esters (FAME) were also processed in duplicate and prepared by base-catalyzed transmethylation of total lipids using sodium methylate solution and boron trifluoride-methanol, according to Bondia-Pons et al. [15]. Then n-hexane with butylated hydroxytoluene (BHT) at 0.02% and NaCl were added. The recovered organic phase was dried with anhydrous sodium sulfate and evaporated to dryness under nitrogen flow and finally redissolved in n-hexane. Gas chromatography analyses were performed on Shimadzu (Kyoto, Japan) gas chromatograph (GC), equipped with a Cp-Sil 88 capillary column (60 m × 0.25 mm I.D., 0.20 μm; Agilent^®^ J&W, Santa Clara, CA, USA) equipped with a Shimadzu flame ionization detector (FID) and a Shimadzu AOC-20i auto-injector. The carrier gas (helium) was set at a constant linear velocity of 20 cm/s. the split-splitless injector was used in split mode with a split ratio of 1:50, injector and detector temperatures of 250 °C and 260 °C, respectively, were used. Oven temperature program was as follows: initial temperature 100 °C for 5 min, increased at 1 °C/min to 215 °C and held at this temperature for 20 min. FAME were identified by comparison with a standard mixture (Sigma 47,885-U Supelco 37 Component FAME Mix; Bellefonte, PA, USA). The fatty acid profile expressed as percentage of total FAs refers to the relative proportions among total FA. The fatty acid expressed as mg/100 g muscle, represents the FA content in the edible portion, and the quantification of each FAME was estimated using the known concentration and signal area of internal standard (tridecanoic acid or C13:0), using the following formula:

FA (mg/100 g muscle) = 100 × [((mg C13:0) × (FAME area)/(C13:0 area))/(g muscle)].

### 2.4. Lipid Quality Indices

The nutritional quality of the fatty acid profile was assessed by considering the following indices: peroxidability index (PI; (1)) [16], atherogenicity index (AI; (2)), thrombogenicity index (TI;(3)) [17].

The ratios of hypocholesterolaemic/hypercholesterolaemic (h/H; (4)) [18]; polyunsaturated/saturated fatty acids (PUFA/SFA; (5)), and the n-3 PUFA /n-6 PUFA (n-3/n-6 (6)) were also calculated, according to the respective Equations, presented below.

(1) PI = (% monoenoic × 0.025) + (% dienoic × 1) + (% trienoic × 2) + (% tetraenoic × 4) + (% pentaenoic × 6) + (% hexaenoic × 8);

(2) AI = (C12:0 + (4 × C14:0) + C16:0)/[(∑MUFA + ∑(n − 6) + ∑(n − 3)];

(3) TI = (C14:0 + C16:0 + C18:0)/[(0.5 × ∑MUFA) + (0.5 × (n − 6)) + (3 × n − 3) + (n − 3/n − 6)];

(4) h/H = [(C18:1n-9 + C18:2n-6 + C18:3n-3 + C20:4n-6 + C20:5n-3 + C22:5n-3 + C22:6n-3)/(C14:0 + C16:0)]

(5) PUFA/SFA = ∑PUFA/∑SFA;

(6) n-3/n-6 = [(∑n-3)/(∑n-6)].

### 2.5. Statistical Analysis

Throughout the results and discussion, the term superiority (expressed as %) was calculated as (maximum value − minimum value)/minimum value, while the term inferiority was calculated as (maximum value − minimum value)/maximum value.

Data were subjected to a one-way ANOVA considering the ocean as the unique variable, using the GLM procedure of SAS (SAS Inst., Cary, NC, USA; version 9.3). Additionally, a second analysis of all data was performed using the TL content in muscle as a covariate. The least square means and relative standard deviation of the mean (RSD) or the standard error of the mean (SEM) are presented in tables.

## 3. Results and Discussion

### 3.1. Lipid Content and Fatty Acid Profile

Data on yellowfin tuna (YFT) harvested in the Atlantic, Indian and Pacific oceans, total lipid (TL), fatty acid (FA) partial sums, FA ratios and the lipid quality indices are presented in Table 2, while the detailed FA profile is shown in Table 3.

The ocean had no significant influence on YFT TL content (*p* = 0.109), total PUFA (*p* = 0.094) and total n-3 PUFA (*p* = 0.146) contents. The YFT harvested in the Indian Ocean displayed significantly higher contents of both SFA and MUFA totals than the Atlantic Ocean counterparts (*p* < 0.05), a superiority of 62.8% and 69.1%, respectively). Whereas the YFT harvested in the Pacific Ocean presented a halfway value of SFA and MUFA sums, not differing significantly from the YFT harvested in the Indian and Atlantic Oceans. The analysis of the FA partial sums proportions revealed that the ocean had no significant influence on total n-6 PUFA (*p* = 0.310), but significantly (*p* < 0.05) influenced the proportion of all other FA partial sums.

It is important to highlight that the analysis of FA partial sums contents and proportions revealed similar results in SFA and MUFA, but total PUFA, n-3 PUFA and n-6 PUFA showed different results when the analysis was focused in contents or in proportions. The ocean had no significant influence in total PUFA and total n-3 PUFA contents (*p* = 0.094 and 0.146, respectively), but displayed a significant influence in their proportions (*p* < 0.001), whereas, the ocean had a significant influence in n-6 PUFA contents (*p* = 0.004), but had no influence on its proportion (*p* = 0.310).

In fish species, the TL contents and fatty acid profile are rather variable between species and even between different specimens of a single species, depending upon different abiotic and biotic factors [19,20,21]. The TL content of YFT white muscle, presented herein (averaged 1.1 g/100 g of muscle), are within the range of values previously presented for this species, ranging between 0.60 and 2.88 g/100 g of muscle [12,22,23]. The YFT white muscle contains minor lipid depots, which are predominantly stored in the liver and the red muscle [21].

The proportions of FA partial sums presented herein, for the YFT white muscle, revealed that total MUFA, total PUFA, total n-3 PUFA and total n-6 PUFA were in the range of values previously presented for the YFT harvested in the Indian Ocean and Arabian Sea [22,24]. The contents of FA partial sums presented herein for the Atlantic YFT (132.3, 55.6 and 171.1 mg/100 g of muscle for SFA, MUFA and PUFA, respectively) were in the range of values previously presented for the species harvested in the Arabian Sea and Indian Ocean [12,24]. Whereas the FA partial sums presented herein for the YFT harvested in the Indian and Pacific Oceans displayed a higher content of total SFA and total MUFA, but a lower content of total PUFA (342.6, 162.7 and 258.3 mg/100 g of muscle for SFA, MUFA and PUFA, respectively) than previously shown for the species harvested in the Arabian Sea and Indian Ocean [12,21,24]. Differences observed herein between different oceans on yellowfin tuna FA partial sums occur even in different fishing areas within a single ocean. It is important to highlight the YFT prime prey species, which include fish, cephalopods, crustaceans from several genera and even some tunicates can change between seasons [25,26,27]. Considering the YFT euriphagic condition, seasonal variations on the predominant prey species and in their TL content and FA profile may contribute considerably to differences in the FA profile of its neutral lipid fraction, which are predominant stored in the liver. Such suggestion is sustained by the study of Sardenne et al. [21], who showed that SFA and MUFA are predominantly stored in the neutral lipid fraction, while PUFA are predominantly used in the biosynthesis of polar lipid fraction. 

Despite the absence of statistically significant differences in the TL contents between YFT origins, the TL content of the YFT harvested in the Indian Ocean presented a superiority of 52.9% and 17.1% over the Atlantic and Pacific counterparts, respectively. Such differences justified a second statistical analysis. Therefore, the FA data were reanalyzed using the TL content in YFT white muscle as a covariate (data not shown) in order to test if the differences observed between different oceans could be explained by loin fattiness. However, differences observed on partial FA sums, FA ratios and lipid quality indices persisted after covariance adjustment to the same TL content. Thus, the nonsignificant differences previously reported on TL contents cannot be responsible for the significant differences observed on FA partial sums, FA ratios and lipid quality indices. The higher FA ratios and lower lipid quality indices presented by the Atlantic YFT allow us to say that the Atlantic YFT offers a healthier fatty acid profile than their Indian and Pacific counterparts. 

In fish, dietary FA are absorbed and stored without important structural modifications as triacylglycerols (neutral lipid fraction or energy reserves), or can also be used in the biosynthesis of complex lipids, as phospholipids, which are allocated into biological membranes (polar lipid fraction or structural lipids) according to cell requirements [28]. Therefore, the FA profile of the polar lipid fraction differs from the profile obtained from the neutral lipid fraction, because cell membranes are dynamic elements that require structural regulation to ensure their biological functions [29].

The FA profile, presented herein, represents the total FA profile, since it encloses the FA from both neutral and polar lipid fractions. In the YFT white muscle 75% and 25% of total FA are representative of the FA present in the polar and neutral lipid fractions, respectively [21]. Changes in the proportion of major lipid fractions (polar/neutral) influence the FA profile, since some FA are predominantly used in the synthesis of phospholipids while others are just stored as triacylglycerols [21]. Independently of the harvesting origin, the YFT loin encloses 28 FA, but only eight FA reach a percentage ≥1% of total FA, namely and by decreasing order: the C22:6n-3 (DHA or docosahexaenoic acid), followed by the C16:0 (palmitic acid), the C18:1n-9 (oleic acid), the C18:0 (stearic acid), the C20:4n-6 (arachidonic acid or ARA), the C20:5n-3 (eicosapentaenoic acid or EPA), the C16:1n-7 (palmitoleic acid), and the C14:0 (myristic acid). Among these eight FA, the stearic and arachidonic acids were the only ones whose contents were not influenced by the ocean. However, these two FA are predominantly used in the phospholipid biosynthesis [21], and that is the probable cause for their constant content in YFT white muscle. Together, the eight prime FA were accountable for 93.8–95.4% of total FA. Within the 28 FA, only six of them were not significantly influenced by the ocean (C18:0, C17:1n-7, C22:1n-9, C20:4n-6, C22:4n-6 and C20:3n-3). The comparison of the YFT from the Atlantic and Pacific Oceans was associated with 18 significant differences; the comparison of the YFT from the Atlantic and Indian Oceans was associated with 16 significant differences, while the comparison of the YFT from the Indian and Pacific Oceans was associated with just nine significant differences. Beyond the analysis of variance, the individual FA were also subjected to the analysis of covariance, after covariance adjustment to the same TL content. The covariance revealed two significant differences on C20:4n-6 and on C20:3n-3 (*p* < 0.001), while the difference observed on C18:2n-6 (linoleic acid) vanished (*p* > 0.05). The same is to say that, differences in the TL content were hiding differences on C20:4n-6 and C20:3n-3, while differences on C18:2n-6 were a consequence of differences in TL content.

Total PUFA was the leading FA group in the Atlantic YFT (48.3% of total FA), whereas the total SFA (averaging 43.7% of total FA) was the most relevant FA group on the YFT from the Indian and Pacific oceans. The Atlantic YFT higher contents of both total PUFA and total n-3 PUFA were a consequence of its higher contents (*p* < 0.001) in DHA, a superiority that was not observed in all other FA belonging to PUFA. DHA is predominantly used in the biosynthesis of phospholipids [21,30], suggesting that the Atlantic YFT presents a higher proportion of polar/neutral lipids. Conversely, the higher SFA contents observed on Indian and Pacific YFT relatively to the Atlantic YFT was a consequence of their higher contents in C14:0, C15:0, C16:0, C17:0, C20:0, C21:0, C22:0 and C23:0. SFA are predominantly stored in triacylglycerols as an energy reserve. However, some SFA (C10:0 and C24:0) were outside of such superiority, and the C18:0 (stearic acid), is used in phospholipid synthesis [21].

To better understand the ocean’s influence on YFT fatty acid profile, future studies must include (1) different fishing seasons, which must overlap in all major oceans, and (2) the characterization of feeding habits are essential.

### 3.2. Nutritional Value

In order to gain insight into the possible health effects of the FA profile presented by YFT, the contents of certain saturated fatty acids (i.e., C12:0, C14:0 and C 16:0) should be taken into account, since they have been evidenced to increase the total serum cholesterol [17]. The PUFA/SFA values for the YFT harvested in the Atlantic, Indian and Pacific Oceans were 1.32, 0.85 and 0.89, respectively (Table 2), being above the recommended value (0.45) [31], indicating that the YFT fatty acid profile may contribute to reducing the risk of cardiovascular, autoimmune and other chronic diseases [17]. PUFA/SFA ratios are used to assess the nutritional value of foods and in fish the values range between 0.50 and 1.62 [32], and the PUFA/SFA values observed herein for YFT are, independently of the origin, inside the range of values observed in fish. Nevertheless, the PUFA/SFA value of the Atlantic YFT was significantly higher than those YFT from the Indian and Pacific Oceans.

Regarding n3/n6, all the values were higher than 4. According to health recommendations, n3/n6 ratios, a balance between anti-inflammatory and proinflammatory metabolic precursors (eicosanoids) should be higher than 4, thereby reducing the incidence of chronic food-related illnesses [33,34], due to the better utilization of n-3 PUFA in the human body [35]. 

As previously observed for the PUFA/SFA and n3/n6 ratios, the h/H ratio of the Atlantic YFT (2.51) is significantly higher than was observed on the Indian and Pacific YFT (1.72 and 1.69), the highest value indicates a lower cholesterolemic impact. The h/H values presented for YFT are in the range of values previously reported on different fish species (1.54–4.83) [32].

The AI values ranged between 0.43 (Atlantic Ocean) and around 0.64 in YFT from the Indian and Pacific Oceans (Table 2). According to health recommendations, AI should be below 1 [17]. This index gives information about the ability of some saturated FA to exhibit proatherogenic effects, while nonsaturated FA are regarded to be anti-atherogenic as they inhibit atheroma formation and diminish the levels of esterified fatty acids, cholesterol, and phospholipids, thereby preventing micro- and macrocoronary events [17]. Moreover, compared with some usual food components (e.g., crops 0.084–0.55; meat 0.165–1.32, dairy products 1.42–5.13) [32], the YFT presents a healthier index. The TI values were also lower in YFT from the Atlantic Ocean (0.253) comparatively to the YFT from the Indian and Pacific oceans (0.425 and 0.378, respectively). The thrombogenic index shows the tendency towards blood clotting and, according to Ulbricht and Southgate [17], they should be lower than 1. TI values for crops, fish, meat, and dairy products were reported as 0.139–0.56, 0.14–0.87, 0.288–1.694, and 0.39–5.04, respectively. [32].

The YFT from the Atlantic Ocean presented higher PI values than both Indian and Pacific counterparts (350 versus 260), such higher oxidative susceptibility is a natural consequence of its FA higher unsaturation index.

The comparison of the YFT harvested in the three major oceans, shows that the Atlantic specimens displayed healthier FA ratios and lipid quality indices, thanks to significantly higher PUFA/SFA, n3/n6 and h/H values and lower indices (AI and TI), being for that reason regarded of superior nutritional quality.

Considering the health-promoting properties of long-chain n-3 PUFA, as EPA and DHA, an evaluation of the FA profile would be incomplete without the evaluation of YFT loin content in these two FA. The total PUFA content in YFT loin ranged between 171.1 and 278.4 mg/100 g of muscle, EPA plus DHA represent 80.5–87.2% of total PUFA. Therefore, with a recommended daily intake of 250 mg of EPA + DHA [36]. A portion of YFT white muscle weighing 100 g from the Atlantic Ocean offers 149.2 mg of EPA plus DHA (59.6% of the recommended daily intake), a similar portion from the Indian Ocean presents 191.8 mg of EPA plus DHA (77.6% of the recommended daily intake), while the equivalent amount from the Pacific Ocean provides 229.4 mg of EPA plus DHA (91.7% of the recommended daily intake).

## 4. Conclusions

Based on the lipid content, YFT may be considered a lean fish. In addition, it is rich in unsaturated fatty acids, in particular PUFA such as ARA, DHA, and EPA. The Atlantic YFT FA profile showed lower contents of SFA and MUFA, but higher contents of PUFA and n-3 PUFA, such differences contributed to higher PUFA/SFA, n3/n6 and h/H and lower AI and TI, making the Atlantic YFT the healthier option.

However, and regardless of the absence of significant differences in TL contents, the Atlantic YFT lower TL content makes it a less beneficial source of EPA plus DHA.

Despite differences observed between different harvest locations, it is possible to conclude that YFT is a healthy source of protein, considering its FA profile and richness in n-3 PUFA.

## Figures and Tables

**Table 1:** Yellowfin tuna weight, fishing period and fishing areas.

Ocean of Origin	Atlantic	Indian	Pacific
Sample size (*n*)	15	15	15
Weight (mean ± standard deviation; kg)	31.7 ± 4.9	50.6 ± 5.8	37.3 ± 12.0
Fishing year	2020	2020	2020
Fishing periods	24 September–26 October	4 April–14 June	20 March–23 April
FAO fishing areas	34	51	71

**Table 2:** Total lipid contents (TL; expressed as g/100 g of flesh), fatty acid partial sums (expressed as mg/100 g of flesh and as % of total FA), fatty acid ratios and lipid quality indices.

	Atlantic		Indian		Pacific		SEM	*p*
TL	0.85		1.30		1.11		0.571	0.109
Partial sums (mg/100 g and (% of total FA))								
Σ SFA	132.3 ^b^	(36.7%)	355.5 ^a^	(43.8%)	329.6 ^a,b^	(43.5%)	59.14	0.020
Σ MUFA	55.6 ^b^	(14.9%)	180.2 ^a^	(19.8%)	145.2 ^a,b^	(17.9%)	33.35	0.033
Σ PUFA	171.1	(48.3%)	238.1	(36.3%)	278.4	(38.6%)	34.24	0.094
Σ n-3 PUFA	150.0	(42.4%)	196.3	(29.6%)	232.1	(32.2%)	28.99	0.146
Σ n-6 PUFA	21.1 ^b^	(5.98%)	41.8 ^a^	(6.77%)	46.3 ^a^	(6.40%)	5.331	0.004
Fatty acid ratios and lipid quality indices								
PUFA/SFA ^1^	1.322 ^a^		0.854 ^b^		0.893 ^b^		0.203	<0.001
n3/n6 ^2^	7.122 ^a^		4.530 ^b^		5.076 ^b^		0.634	<0.001
h/H ^3^	2.510 ^a^		1.724 ^b^		1.693 ^b^		0.312	<0.001
AI ^4^	0.436 ^b^		0.643 ^a^		0.640 ^a^		0.095	<0.001
TI ^5^	0.253 ^b^		0.425 ^a^		0.378 ^a^		0.075	<0.001
PI ^6^	350.6 ^a^		250.9 ^b^		269.0 ^b^		44.52	<0.001

Different superscripts in the same row diverge significantly (*p* < 0.05); SEM stands for standard error of mean. SFA stands for saturated fatty acids. MUFA stands for monounsaturated fatty acids. PUFA stands for polyunsaturated fatty acids. ^1^ PUFA/SFA was calculated as Σ PUFA/Σ SFA. ^2^ n3/n6 was calculated as Σ n3 PUFA/Σ n6 PUFA. ^3^ h/H stands for hypocholesterolaemic/hypercholesterolaemic ratio. ^4^ AI stands for atherogenicity index. ^5^ TI stands for thrombogenicity index. ^6^ PI Stands for peroxidability index.

**Table 3:** Detailed fatty acid profile of yellowfin tuna (*Thunnus albacares*) loin from the Atlantic, Indian and Pacific Oceans (expressed as % of total FA).

	Ocean			Statistics	
	Atlantic	Indian	Pacific	RSD	*p*
C10:0	0.34 ^a^	0.33 ^a^	0.08 ^b^	0.324	0.004
C14:0	1.13 ^b^	1.56 ^a,b^	1.85 ^a^	0.488	<0.001
C15:0	0.42 ^b^	0.65 ^a^	0.65 ^a^	0.128	<0.001
C16:0	23.1 ^b^	29.3 ^a^	28.6 ^a^	2.545	<0.001
C17:0	0.72 ^b^	0.88 ^a^	0.99 ^a^	0.141	<0.001
C18:0	10.5	10.3	10.2	1.172	0.722
C20:0	0.14 ^c^	0.23 ^b^	0.34 ^a^	0.080	<0.001
C21:0	0.03 ^c^	0.05 ^b^	0.07 ^a^	0.015	<0.001
C22:0	0.10 ^c^	0.15 ^b^	0.24 ^a^	0.057	<0.001
C23:0	0.05 ^c^	0.07 ^b^	0.10 ^a^	0.022	<0.001
C24:0	0.22 ^b^	0.23 ^b^	0.35 ^a^	0.043	<0.001
C16:1 n-7	1.30 ^b^	2.88 ^a^	3.32 ^a^	0.974	<0.001
C17:1 n-7	0.07	0.11	0.06	0.053	0.058
C18:1 n-11	0.08 ^b^	0.13 ^a^	0.12 ^a^	0.031	<0.001
C18:1 n-9	12.5 ^b^	15.8 ^a^	13.1 ^b^	2.864	0.007
C20:1n-9	0.42 ^b^	0.48 ^a,b^	0.63 ^a^	0.206	0.022
C22:1 n-9	0.04	0.05	0.05	0.016	0.329
C24:1 n-9	0.51 ^a,b^	0.40 ^b^	0.58 ^a^	0.119	<0.001
C18:2 n-6	0.90 ^b^	0.99 ^a,b^	1.05 ^a^	0.150	0.022
C18:3 n-6	0.03 ^b^	0.06 ^a^	0.06 ^a^	0.016	0.001
C20:2 n-6	0.22 ^b^	0.32 ^a^	0.23 ^b^	0.050	<0.001
C20:3 n-6	0.10 ^b^	0.18 ^a^	0.18 ^a^	0.025	<0.001
C20:4 n-6	4.75	5.25	4.89	1.385	0.600
C22:2 n-6	0.02	0.02	0.03	0.008	0.482
C18:3 n-3	0.19 ^b^	0.27 ^a,b^	0.32 ^a^	0.102	0.004
C20:3 n-3	0.09	0.09	0.09	0.027	0.871
C20:5 n-3	3.72 ^a,b^	3.34 ^b^	4.03 ^a^	0.507	0.003
C22:6 n-3	38.4 ^a^	25.9 ^b^	27.8 ^b^	4.994	<0.001

Different superscripts in the row are associated with significant differences (*p* < 0.05); RSD—stands for relative standard deviation; SFA stands for saturated fatty acids; MUFA stands for monounsaturated fatty acids; PUFA stands for polyunsaturated fatty acids.
